# Ultraviolet Lasers Realized via Electrostatic Doping Method

**DOI:** 10.1038/srep13641

**Published:** 2015-09-01

**Authors:** X. Y. Liu, C. X. Shan, H. Zhu, B. H. Li, M. M. Jiang, S. F. Yu, D. Z. Shen

**Affiliations:** 1 State Key Laboratory of Luminescence and Applications, Changchun Institute of Optics, Fine Mechanics and Physics, Chinese Academy of Sciences, Changchun 130033, China; 2 School of Physical Engineering, Zhengzhou University, Zhengzhou 450052, China; 3 State Key Laboratory of Optoelectronic Materials and Technologies, School of Physics and Engineering, Sun Yet-Sen University, Guangzhou 510275, China; 4 Department of Applied Physics, The Hong Kong Polytechnic University, Hung Hom, Kowloon, Hong Kong, China

## Abstract

*P*-type doping of wide-bandgap semiconductors has long been a challenging issue for the relatively large activation energy and strong compensation of acceptor states in these materials, which hinders their applications in ultraviolet (UV) optoelectronic devices drastically. Here we show that by employing electrostatic doping method, hole-dominant region can be formed in wide bandgap semiconductors, and UV lasing has been achieved through the external injection of electrons into the hole-dominant region, confirming the applicability of the *p*-type wide bandgap semiconductors realized via the electrostatic doping method in optoelectronic devices.

Wide bandgap semiconductors have a variety of potential applications, including light-emitting devices, short-wavelength lasers, etc^1^,^2^,^3^. In many cases, to realize these applications, *p*-type doping of the semiconductors is a fundamental step. Although there has been steady progress in the *p*-type doping of III-nitride semiconductors^4^,^5^, *p*-type doping of wide bandgap semiconductors is still one of the most challenging issues that hinder the realization of the above mentioned devices^6^. It is accepted that the *p*-type doping of wide bandgap semiconductors has two major obstacles. One is the relatively large activation energy of acceptors, and the other is the relatively strong compensation. For example, the activation energy of magnesium acceptor in gallium nitride is about 200 meV^7^, and that of nitrogen in another important wide bandgap semiconductor, zinc oxide (ZnO), is around 170 meV^8^. Both of these activation energies are well above the thermal energy *k*_B_*T* (which is 26 meV at room temperature, where *k*_B_ is Boltzmann constant and *T* is the Kelvin temperature), which impedes drastically the release of holes from the acceptors. As for the compensation, it means that when acceptors are doped into the host materials, besides the residual donors in the host materials, donor-related defects will be introduced to lower the total energy of the system^9^. These donor-related defects will electrically compensates the doped acceptors, making the *p*-type doping ineffective or even impossible. The aforementioned high activation energy and strong compensation hinder drastically the realization of *p*-type doping in wide bandgap semiconductors, thus limiting their potential applications in light-emitting devices and laser diodes.

It is accepted that when a negative bias is applied onto the larger bandgap semiconductor side of a heterojunction, holes in the smaller bandgap semiconductor will drift to and accumulate at the interface of the heterojunction, while electrons will be depleted^10^,^11^. As a result, holes will dominate the carrier transportation along the interface, which means that *p*-type doping at this region will be realized. The above process is called electrostatic doping, which has been elucidated in graphene, semiconducting carbon nanotubes, WSe_2_, etc, and light-emitting devices, photodetectors, and photovoltaic devices have been realized via this method^12^,^13^,^14^,^15^,^16^,^17^,^18^,^19^. Note that all the above materials have relatively narrow bandgap. One can speculate that if such an electrostatic doping method could be used for the *p*-type doping of wide bandgap semiconductors, the major obstacles of large activation energy and strong compensation will be circumvented, then reliable light-emitting devices and even lasers may be realized. However, such a report is still absent to date.

In this paper, by employing ZnO as a representative, we show that by employing the electrostatic doping method, quasi *p-n* junctions have been formed, and by applying forward bias to the quasi *p-n* junctions, ultraviolet (UV) lasing has been observed. None report on lasers realized via electrostatic doping route can be found before to the best our knowledge.

## Results

**Figure 1:**
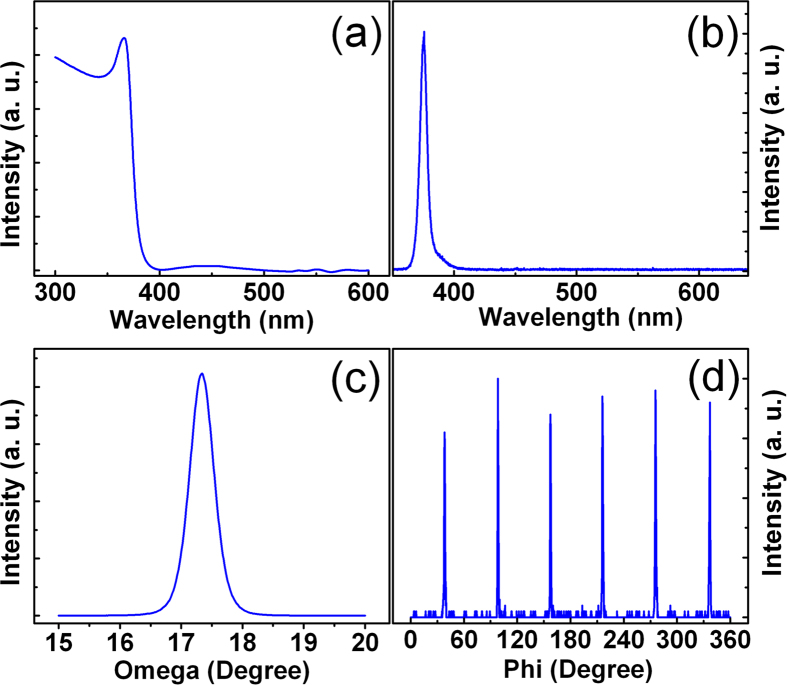
Room temperature absorption spectrum (a), PL spectrum (b), x-ray rocking curve (c), and phi-scan pattern (d) of the ZnO films.

The electron concentration and Hall mobility of the ZnO film employed for the electrostatic doping study is about 6.0 × 10^16^ cm^−3^ and 56 cm^2^ V^−1^ s^−1^, respectively. The structural and optical characterizations of the film are shown in Fig. 1. The absorption spectrum of the film shown in Fig. 1a displays a dominant peak at 360 nm, which corresponds to the excitonic absorption of ZnO. Figure 1b shows the photoluminescence (PL) spectrum of the film, in which a sharp emission at around 380 nm, typically attributed to the near-band-edge (NBE) emission of ZnO, can be observed, while the deep-level related emission is almost undetectable. The x-ray rocking curve of the ZnO film is shown in Fig. 1c, where a peak at 17.3° is visible. Figure 1d shows the phi-scan of the film, in which six peaks separated by 60° can be observed, indicating the six-fold symmetry of the ZnO film.

**Figure 2:**
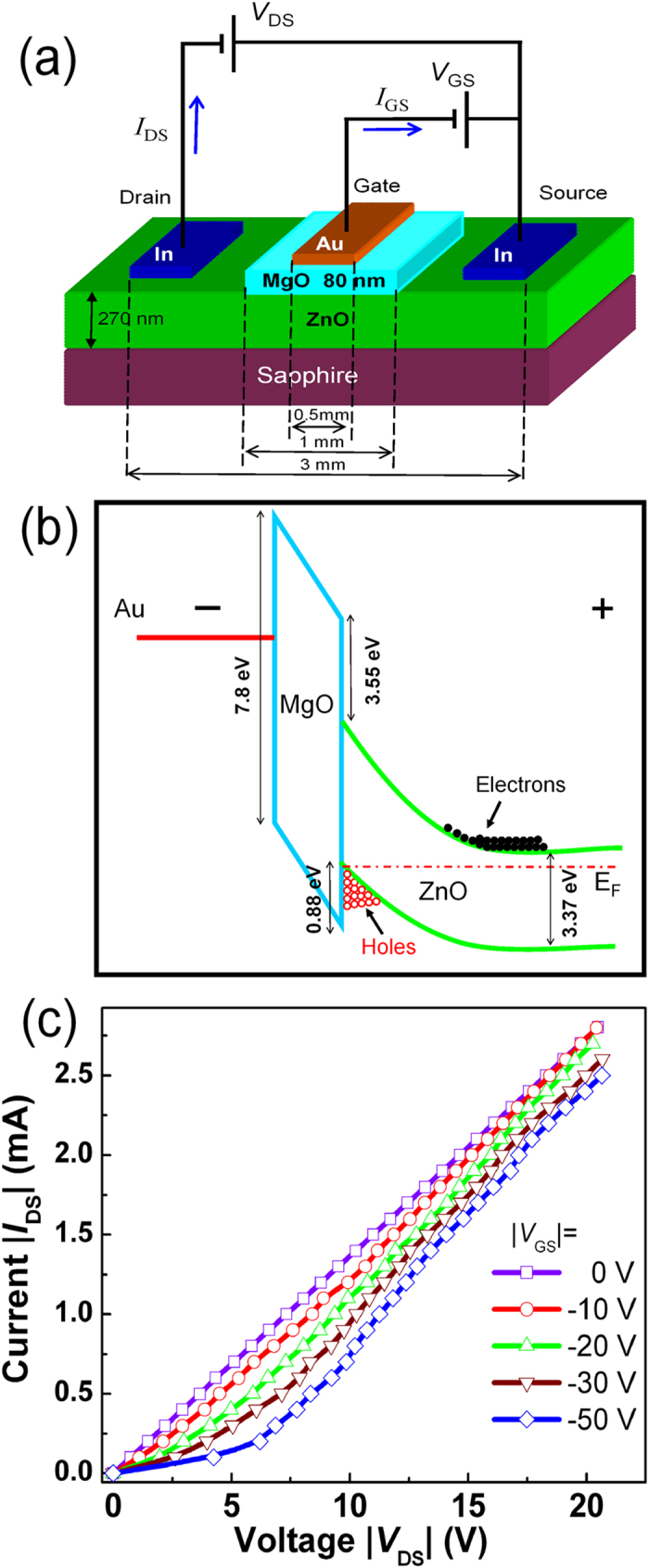
(**a**) Schematic diagram of the designed structure; (**b**) Band alignment of the Au/MgO/ZnO junction under reverse bias showing the formation of hole-dominant conduction area in the structure; (**c**) The dependence of the current between the source and drain electrode of the structure on the voltage applied on these two electrodes.

Based on the ZnO layer, Au/MgO/ZnO structures have been constructed to test the idea of electrostatic doping. A schematic illustration of the structure is shown in Fig. 2a, in which a Au bar was coated onto the MgO layer acting as a gate electrode, and two In bars were deposited onto the ZnO layer acting as the source and drain electrodes. To show the mechanism for the electrostatic *p*-type doping process, the bandgap diagram of the Au/MgO/ZnO structure under reverse bias is illustrated in Fig. 2b. When a negative bias is applied onto the Au/MgO/ZnO structure, both the conduction and valance band of the ZnO at the MgO/ZnO interface will be bent downward. Then the electrons will be repelled, and when the bias is large enough, holes may concentrate in the top layer of the ZnO film. That is, the conduction in some area of the ZnO film may be inverted. As a result, the conductivity of the region near the interface may be dominated by holes, that is, *p*-type conduction can be induced by the negative bias voltage. Considering that the conductivity of the other region is still electron-dominant, quasi *p-n* junctions will be formed. To confirm the formation of such quasi *p-n* junctions, the current-voltage (*I* - *V*) curves of the structure have been measured, as shown in Fig. 2c. With zero gate voltage, the *I*−*V* curve between the source and the drain contact, indicated by the square symbols, does not show any rectification behavior. When the gate voltage is increased gradually, noticeable rectification behaviors are observed. Note that when the gate voltage reaches −50 V, the *I*−*V* curve between the source and drain contact shows a significant rectification effect with a turn-on voltage of about 5.1 V, which reveals the formation of quasi *p-n* junctions in the structure due to the electrostatic doping.

**Figure 3:**
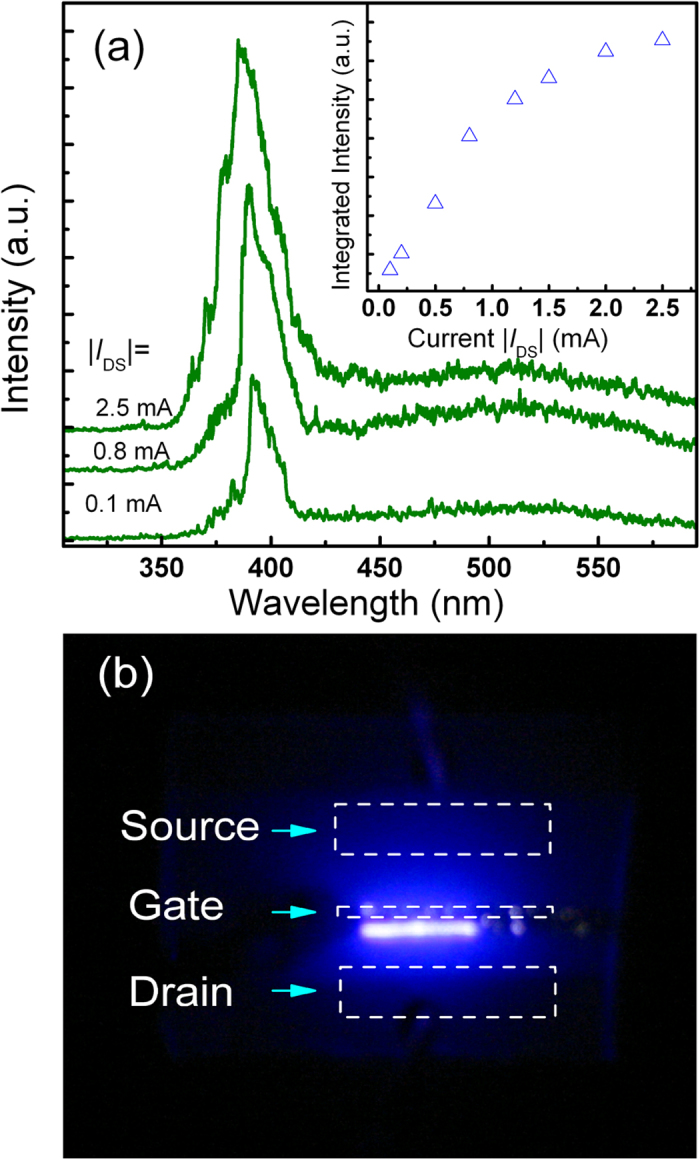
(**a**) EL spectra measured from the top surface of the Au/MgO/ZnO structure. In the measurement, |*V*_*GS*_| is set as −50 V and |*I*_*DS*_| varies from 0.1 to 2.5 mA. The inset shows the dependence of the integrated emission intensity on |*I*_*DS*_|; (**b**) Far-field emission image recorded from the top surface of the Au/MgO/ZnO structure.

When a forward bias voltage is applied onto the quasi *p-n* junctions formed via the electrostatic doping method, the electrons in the undoped region will be injected into the inversion region, and recombine with holes there, then emission may be obtained. Experimentally, by fixing the gate voltage at −50 V, when a bias is applied onto the source and the drain contact, obvious emission has been observed from the top surface of the structure, the spectrum of which is shown in Fig. 3a. It shows a dominant peak at about 390 nm, which is the typical NBE emission of ZnO, while the deep-level related emission is weak. The dependence of the integrated emission intensity of the device recorded from the top surface on the injection current between the source and drain contact (|*I*_DS_|) is shown in the inset of Fig. 3a. It reveals that the emission intensity increases with the injection current, and it saturates gradually at larger injection current. Figure 3b plots the far-field emission image recorded from the top surface of the Au/MgO/ZnO structure. Obvious blue emission can be clearly observed along the gate electrode, which verifies that the radiative recombination between electrons and holes occurs mainly in the ZnO film near the gate electrode, confirming that the validity of the electrostatic doping route.

**Figure 4:**
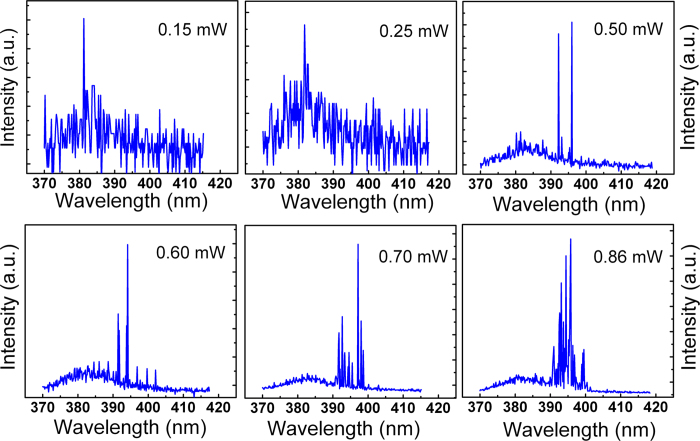
PL spectra of the ZnO film excited by a 266 nm laser.

It is accepted that due to the relatively large exciton binding energy of ZnO (60 meV), lasing can be realized via an exciton-exciton scattering mode, and the threshold of which can be over two orders of magnitude smaller than that realized via an electron-hole pair mode^20^. Therefore, it is expected that low-threshold lasing may be realized in ZnO. Actually, many lasing emissions have been observed in ZnO films, nanostructures and powders^21^,^22^,^23^,^24^,^25^,^26^,^27^, To show the feasibility of the electrostatic doping in ZnO based lasers, optically pumped lasing has been measured on the ZnO layer employed as active layer of the electrostatic doping, and the spectra are shown in Fig. 4. When the pumping power is 0.15 mW, although the emission of the ZnO layer is weak, a broad band at around 380 nm can also be observed, which corresponds to the NBE emission of ZnO, and the full-width at half maximum (FWHM) of the emission is about 10.5 nm. When the excitation power is increased to 0.50 mW, some sharp peaks superimposed on the low-energy side of the broad band, are visible in the spectrum, and the FWHM of the sharp peaks is around 0.3 nm. With further increasing the excitation power, more such sharp peaks appear, and the intensity of the peaks increases greatly. It is visible that the PL intensity increases gradually then abruptly with the excitation power. The appearance of sharp peaks at elevated excitation pumping power reveals that lasing has been realized in the ZnO layer.

**Figure 5:**
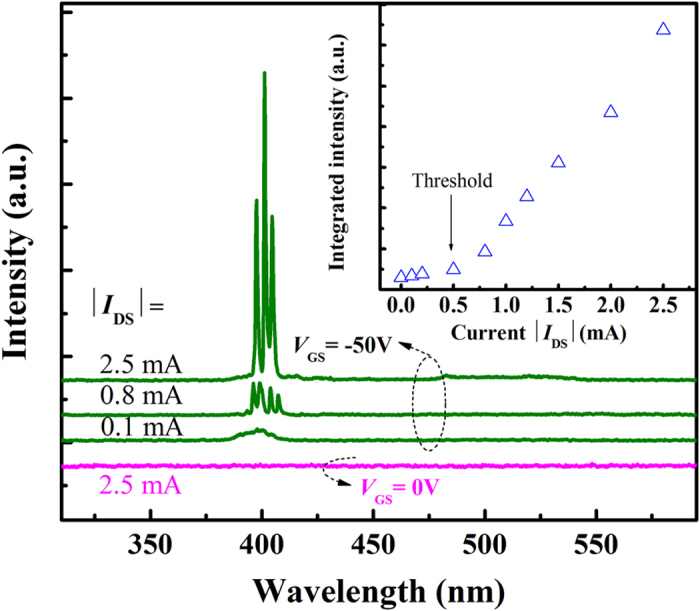
EL spectra measured from the edge of the Au/MgO/ZnO structure. In the measurement, |*V*_GS_| is set as −50 V and |*I*_DS_| varies from 0.1 to 2.5 mA. The emission spectrum of the device when |*V*_GS_| is set as zero and |*I*_DS_| as 2.5 mA is also illustrated in the figure, and the inset shows the dependence of the integrated emission intensity recorded from the side of the device on |*I*_DS_|.

The optically pumped lasing observed in the ZnO films suggests that the films can be employed as an arena for testing the electrically lasing via the electrostatic doping method. To this end, by fixing the gate bias at −50 V, the voltage between the source and drain electrodes have been varied, the emission is recorded from the edge of the structure, the results of which are shown in Fig. 5. When |*I*_DS_| is 0.1 mA, there appears a broad emission band with peak wavelength located at ~395 nm and the corresponding FWHM is ~28 nm. This emission is very similar in shape and position with the emission detected from the top surface of the devices shown in Fig. 3a. With increasing |*I*_DS_| to 0.8 mA, a few sharp peaks emerge from the broad emission spectrum. By further increasing |*I*_DS_| to 2.5 mA, more sharp peaks appear. The dependence of the integrated emission intensity on |*I*_DS_| is shown in the inset of Fig. 5. It is noted that when |*I*_DS_| is less than 0.5 mA, the emission intensity increases linearly with |*I*_DS_|, while the emission intensity increases abruptly when |*I*_DS_| is larger than 0.5 mA. The emission intensity can be increased by more than 32 times, and the width of the emission reduced by a factor of over 20 for |*I*_DS_| increases from 0.1 to 2.5 mA. The observation of kink point in the light-current curve and the reduction in the FWHM of the emission spectra suggested that lasing emission has been realized in the Au/MgO/ZnO structures^28^,^29^. We note that if |*V*_GS_| is set as zero, electrostatic doping process will not occur under such case, no emission can be detected from the structure, as indicated in Fig. 5, which confirm that the lasing is resulted from the holes realized via the electrostatic doping process. To the best of our knowledge, none report on lasers realized via electrostatic doping route can be found before. Notably, the threshold of 0.5 mA is the smallest value ever reported for UV lasers^24^. The electrically pumped lasing phenomenon realized in the Au/MgO/ZnO structure reveals the effectiveness of the electrostatic doping method.

**Figure 6:**
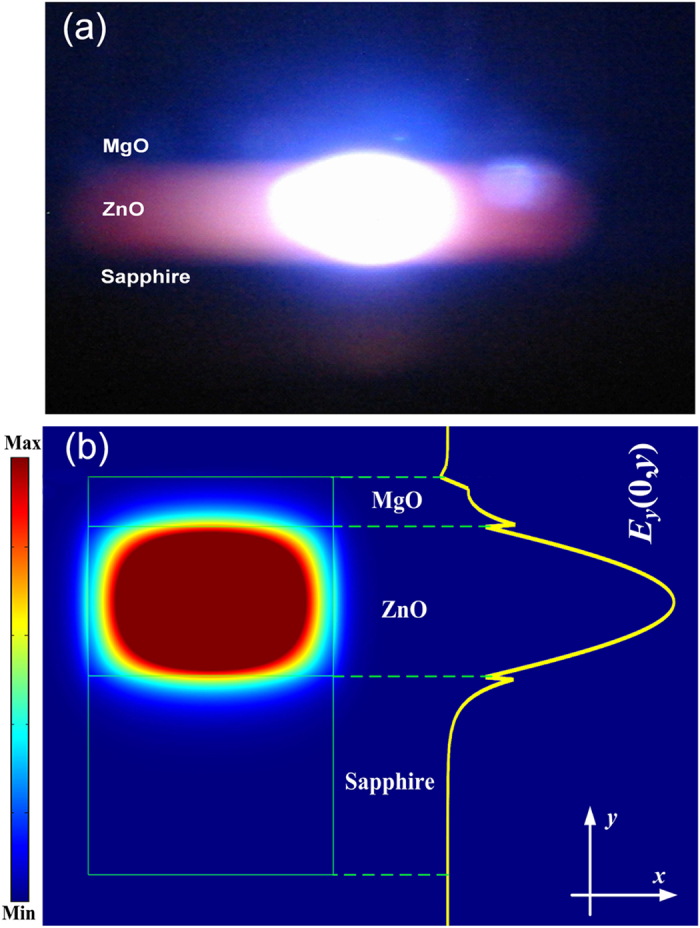
(**a**) Far-field emission image observed from the edge of the Au/MgO/ZnO structure; (**b**) The simulated electromagnetic field distribution of the emission from the edge of the Au/MgO/ZnO structure.

**Figure 7:**
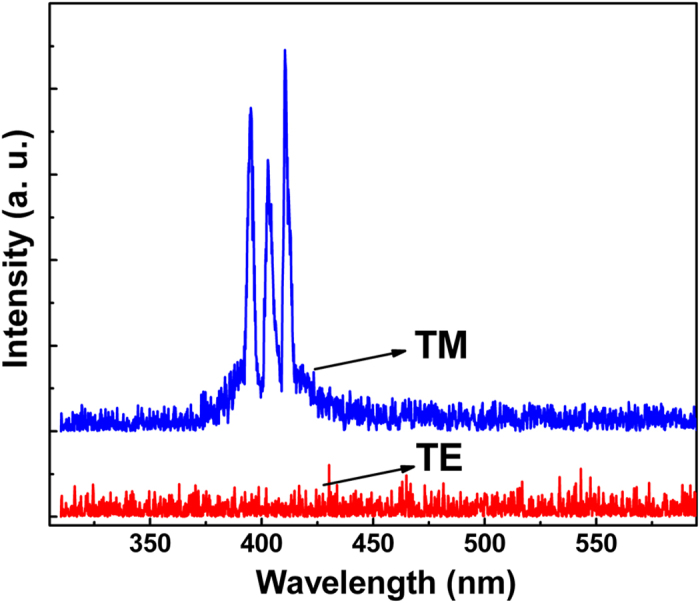
The emission polarization of the lasing obtained from the Au/MgO/ZnO structure via the electrostatic doping method.

Figure 6a shows the far-field emission image observed from the edge of the Au/MgO/ZnO structure. A bright elliptical-shaped spot can be detected from the ZnO layer. The electromagnetic field distribution of the emitted light in the Au/MgO/ZnO structure has been analyzed by finite-different time domain (FDTD) method, as shown in Fig. 6b. One can see that most of the emission energy is confined in the ZnO layer, which agrees well with the far field emission image shown in Fig. 6a. The polarization of the lasing has been studied by measuring the transverse electric (TE) and transverse magnetic (TM) emission spectra of the structure, as shown in Fig. 7. One can see from the figure that a strong polarization of the lasing emission can be observed, and the emission is believed to be E// <0001> polarized from the dominant TM optical gain in the Au/MgO/ZnO structure.

## Discussion

**Figure 8:**
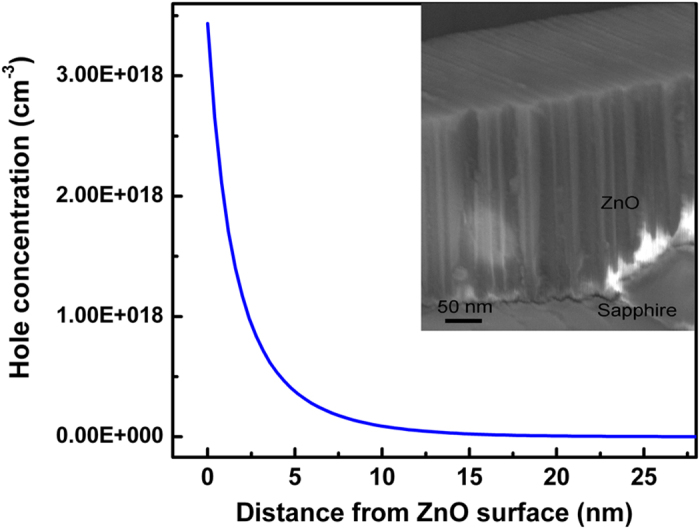
The carrier distribution in the ZnO film when a negative bias is applied onto the gate electrode, and the inset shows the cross-sectional scanning electron microscope image of the ZnO layer.

The mechanism for the electrically pumped lasing observed in the Au/MgO/ZnO structures via the electrostatic doping method can be understood as follows: When a negative bias is applied onto the gate electrode, the distribution of the potential, *Ψ*(*x*), in the ZnO film can be calculated by solving the Poission’s equation:


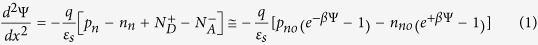


where *β* = *q*/*kT*, *q* is the elemental charge, *k* is the Boltzman constant, *T* is the temperature, and *ε*_s_ (=11.9 × 8.85 × 10^−14^ F/cm) is the permittivity of ZnO. In Eq. 1, we have assumed that the donor concentration *N*_D_^+^ ≈ *n*_i_, *n*_no_ equals to 6 × 10^16^ cm^−3^, the intrinsic electron concentration of ZnO, *n*_i_, equals to 6.0 × 10^−9^ cm^−3^, (*L*) = ∂(*L*)/∂*x* = 0, and *L* (=270 nm) is the thickness of the ZnO layer. Hence, for the ZnO layer to be completely depleted, it is required that the surface potential *ψ* reaches 3.2 V. When the reverse bias is over 3.2 V, the thickness of the inversion layer is larger than 5 nm and the maximum hole concentration can reach 5 × 10^18^ cm^−3^, as shown in Fig. 8a. When a bias is applied between the source and drain electrode of the structure, electrons will be injected into the hole-dominant region, and emission will be observed there, as indicated in Figs. 3 and 5. The cross-sectional morphology of the ZnO is shown in the inset of Fig. 8. One can see that the ZnO layer is composed of packed nanocolumns with average diameter and height of about 20 nm and 270 nm, respectively. Considering that the refractive indices of ZnO and air at around 390 nm is 2.45 and 1.0^27^, respectively, then because of the large refractive index difference between ZnO and air, the ZnO layer underneath the Au bar allows the longitudinal propagation of waveguide modes to achieve optical amplification, and once the gain in the ZnO layer exceeds the loss, lasing will be detected^22^,^27^,^30^,^31^,^32^.

From the above investigation, the mechanism to achieve lasing emission from the Au/MgO/ZnO structure under electrical excitation can be summarized as follows: For the increase of |*V*_GS_| from zero, quasi *p*-*n* junctions will be established underneath the Au gate electrode. However, due to the limited amount of electrons and holes, only weak spontaneous emission is observed from the structure when the |*I*_DS_| is small. Further increase of |*I*_DS_| will increase the generation of holes and the external injection of electrons inside the quasi *p*-*n* junctions. As a result, the inversion region underneath the gate electrode will act as a gain medium to support amplification of spontaneous emission. As the ZnO layer composed by packed nanocolumns can provide transverse confinement of propagation modes as well as longitudinal random distribution of optical feedback, the excitation of spontaneous emission can eventually sustain random lasing action. As a result, lasing emission is observed from the edge of the ZnO structure and the lasing modes are random and TM polarized.

In summary, ZnO quasi *p-n* junctions have been formed by an electrostatic doping method, and UV lasing has been obtained from the structures at room-temperature. The threshold of the laser (about 0.5 mA) is amongst the best value ever reported for a UV laser. We think the electrostatic doping route may be applicable to other wide bandgap semiconductors, thus the results reported in this paper may provide an alternative promising route to efficient optoelectronic devices by circumventing the two major obstacles of large activation energy and strong compensation during the *p*-type doping of wide bandgap semiconductors, thus may promise high-performance ultraviolet optoelectronic devices.

## Methods

For the electrostatic doping, a ZnO/MgO heterojunction has been constructed on a commercially available Al_2_O_3_ 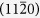 template. Note that the ZnO layer was deposited using a VG V80H plasma-assisted molecular beam epitaxy system. High-purity (6N) elementary zinc and radical O, produced in a plasma cell working at 350 W, were employed as the precursors for the growth of ZnO. The pressure and temperature during the growth process were fixed at 2 × 10^−3^ Pa and 600 °C, respectively. The thickness of the ZnO layer is 270 nm. After that, a 80 nm MgO layer was deposited onto the ZnO layer by magnetron sputtering. Au and In electrode were employed as the contacts to the MgO and ZnO layer, respectively. The crystalline properties of the ZnO layers were evaluated by a Bruker-D8 Discover x-ray diffractometer with Cu Kα (λ = 1.54 Å) as the radiation source. The electrical properties of the ZnO layer were measured using a Lakeshore 7707 Hall measurement system. The thicknesses of the layers were determined by a Hitachi S4800 scanning electron microscope. PL spectra of the ZnO layer were recorded using a JY-630 micro-Raman spectrometer employing the 325 nm line of a He-Cd Laser as the excitation source. Electroluminescence (EL) measurement was carried out in a Hitachi F4500 spectrometer, and a continuous bias was applied onto the gate electrode, while a pulsed bias on the source and drain electrode. For the optically pumped lasing of the ZnO layer, the 266 nm line of the fourth harmonic of a YAG:Nd pulse laser was employed as the excitation source. Note that all the measurements were performed at the room temperature. FDTD method has been used to simulate the electromagnetic field distribution in the devices.

## Additional Information

**How to cite this article**: Liu, X. Y. *et al*. Ultraviolet Lasers Realized via Electrostatic Doping Method. *Sci. Rep*. **5**, 13641; doi: 10.1038/srep13641 (2015).
